# Supplementary material for: Sex-based influence of morphological and physical attributes on serve performance in adolescent volleyball players
Source: PeerJ. 2025 Sep 8;13:e19992. doi: 10.7717/peerj.19992 (PMC12424611; doi:10.7717/peerj.19992)
Supplement: Supplemental Information 2 [file peerj-13-19992-s002.docx]

**Supplementary file**

**10-m linear sprint test**

The 10-meter linear sprint test, was assessed using a series of paired photocells (Globus, Mictogate, Bolsano, Italy). The player started from a standing position with the front foot placed 0.2 m behind the first photocell beam. The photocells were placed at 0.2 m height at the starting position, with a marker for the front foot placed 0.5 m behind this position, and at 1 m height at 10 m. Upon hearing the evaluator's whistle, the player ran as fast as possible. The time taken to cover the distance was recorded in seconds (s). The player performed three attempts, and the best one was retained for analyses. The attempts were separated by 4-5 min recovery intervals.

**Hand grip test**

A handgrip dynamometer (Model 5030L1, Lafayette Instrument, USA) was used to measure hand grip force (Roberts et al., 2011). The participant was asked to hold the dynamometer with the dominant hand, with the arm at a right angle and the elbow at the side of the body. The dynamometer handle was adjusted as necessary - the base should rest on the first metacarpal (heel of the palm), while the handle should rest on the middle of the four fingers. Once ready, the participant squeezes the dynamometer with maximum isometric effort, which is held for approximately 5 seconds. No other body movements are permitted. The result was recorded as kilograms taken from the digital display of the dynamometer to the nearest 0.1 kg. The value was reset to zero before each subsequent measurement. The hand grasp of the dynamometer is comfortable and adjustable to the hand size of the participant. Two attempts were allowed, interspersed by 3 min of passive recovery, and the best result was recorded.

***Trunk flexibility assessment***

Before the sit and reach test (Castro-Piñero et al., 2009), a warm-up with stretching exercises was performed for the muscle groups involved in the evaluation and a demonstration was realized for participants. Sitting in front a box, legs tight and knees straight, the participant flexed the trunk slowly with both arms outstretched, reached as far as possible with his fingers the above box. Participant knees straight was maintained by the experimenter. The participant performed two attempts, and the best result (in cm) was used for subsequent analysis.
